# InVEST Model and Landscape Indices Reveal Habitat Quality Degradation From Land Use Changes in South China

**DOI:** 10.1002/ece3.72719

**Published:** 2025-12-17

**Authors:** Xiaojun Wang, Guangxu Liu, Hong Jia

**Affiliations:** ^1^ School of Geography and Environmental Engineering Gannan Normal University Ganzhou Jiangxi China; ^2^ Institute of Geology and Geophysics Chinese Academy of Sciences Beijing China; ^3^ Key Laboratory of Environmental Change and Natural Disaster, Ministry of Education Beijing Normal University Beijing China; ^4^ Institute of Geographical Science Taiyuan Normal University Jinzhong Shanxi China

**Keywords:** habitat quality, land use, landscape indices, South China, spatial autocorrelation

## Abstract

The intensification of human activities has degraded the ecological environment of Hunan Province in South China. Existing studies on the province's habitat quality (HQ) primarily focus on specific landscapes or geomorphic zones, lacking quantitative analysis of the contribution of land use and land cover change (LULC) to HQ changes, as well as the application of landscape indices to characterize HQ dynamics. This study evaluated HQ changes in Hunan Province from 2000 to 2023, landscape indices, and spatial autocorrelation analysis. The results indicate that LULC was dominated by forestland (62.16%–62.6%) and cultivated land (27.82%–28.83%), and HQ was mainly categorized as high‐quality (40.73%–42.23%) and medium‐quality (24.14%–25.1%), with them concentrated in the mountainous regions, central plains, and lakeshores of western, southern, and eastern Hunan. For both LULC and HQ, landscape fragmentation, instability, and heterogeneity increased over the study period. LULC changes were primarily driven by the mutual conversion between cultivated land and forestland (−30.26% to +29.86%) and the expansion of built‐up land (mainly converted from other land types); HQ degradation (high to low‐quality) was most frequent during the 2015–2020 period. Specifically, the conversion of cultivated land to forestland had a positive effect on the total HQ (+29.86%), whereas the conversion of forestland to cultivated land reduced HQ (−30.26%); the conversion between built‐up land and other land types exerted the most significant effect on the average HQ (> 4.93%). A strong positive spatial autocorrelation was observed between LULC and HQ indices, with significant clustering in mountainous and urban regions. This study demonstrates that LULC changes, particularly irreversible urbanization, drive HQ degradation by increasing landscape fragmentation and heterogeneity. To mitigate ecological fragmentation, sustainable land‐use planning must prioritize forestland conservation, cultivated land protection, and rational urban expansion. Additionally, integrating LULC monitoring with HQ assessments is critical for balancing socioeconomic development and ecological integrity in South China.

## Graphical abstract

Evaluated the habitat quality changes in Hunan Province from 2000 to 2023. Applying landscape index to evaluate the habitat quality changes. Quantified the contribution of different land use and land cover change (LULC) changes to habitat quality. Spatial correlation is used to analyze the relationship between LULC and habitat quality.

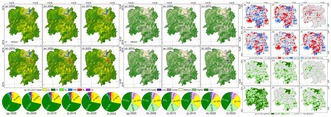

## 1 Introduction

The 15th Sustainable Development Goal (SDG 15) explicitly mandates the protection, restoration, and promotion of sustainable use of terrestrial ecosystems, sustainable forest management, combating desertification, halting and reversing land degradation, and curbing biodiversity loss (ESCAP 2022). Biodiversity plays a pivotal role in sustaining both natural ecosystems and human societies (Robinson et al. 2024). Consequently, protecting biodiversity and rationally utilizing natural environmental resources are critical for supporting socioeconomic development, tourism industry growth, and human physical and mental well‐being (Fisher et al. 2023; Wang, Xiong, et al. 2024). However, with the intensification of human activities, such as rapid urbanization and environmental pollution, the adverse impact on biodiversity, surface ecosystems, and ecological security has become increasingly prominent (Wang, Liu, Xiang, et al. 2022; Wang, Zhong, Wang, et al. 2022; Wang, Liu, Zhang, et al. 2023). The expansion of human‐induced activities directly drives the destruction of natural habitats and exacerbates biodiversity loss (Yang, Xu, et al. 2024). Against this backdrop, conducting habitat surveys, assessing habitat quality, and implementing biodiversity conservation strategies from multitemporal and multispatial perspectives have garnered widespread academic and practical attention (Guo, Niu, et al. 2023).

The Integrated Valuation of Ecosystem Services and Tradeoffs (InVEST) model serves as a pivotal tool for ecosystem service assessment (Jia et al. 2024), enabling the quantification and evaluation of multiple ecosystem services (e.g., water yield, soil conservation, carbon storage, and habitat quality) across diverse global environmental contexts (Wang, Liu, Lin, et al. 2022; Dong et al. 2023; Li et al. 2025). Specifically, the application of the InVEST model in habitat quality assessment has been extensively documented across various research regions, driving factor analyses, and methodological frameworks. For instance, it has been used to study wetland ecosystem characteristics, biodiversity protection, habitat quality evolution under different development scenarios, and the impacts of human activities (e.g., agriculture) on arid ecosystems in countries such as India, South Korea, Türkiye, Lithuania, West African nations, and Mexico (Choudhary et al. 2021; Hong et al. 2021; Ersoy Tonyaloğlu 2025; Cornejo‐Denman et al. 2023). In China, InVEST‐based habitat quality research focuses on urban/mining areas, watershed wetlands, and nature reserves, with urban agglomerations (intensely affected by human activities) being the most studied. For instance, China's major urban agglomerations all exhibit habitat quality degradation. The Guangdong Hong Kong Macao Greater Bay Area shows a peripheral‐to‐central decline in habitat quality (with significant degradation between 1995 and 2020 and projected further decline by 2030), while Shenzhen (within this bay area) has more pronounced degradation due to rapid urbanization (Wu et al. 2024; Zhang, Fan, et al. 2024; Wang, Gu, and Yu 2023; Zhao et al. 2023). Mining areas also face severe habitat quality degradation driven by mining and urban construction activities (Chen et al. 2024). In watershed wetlands, human activities like aquaculture and reclamation have caused rapid habitat quality decline, in contrast to the relatively high quality of natural wetlands (Xing et al. 2023). Even nature reserves are threatened. For instance, Poyang Lake National Nature Reserve has seen declining habitat quality (impacting the population of overwintering migratory birds), and multiple nature reserves in Hubei Province have experienced reduced water areas (due to the expansion of cultivated, built‐up land, and forestland) and a subsequent overall decline in habitat quality, accompanied by increased spatial heterogeneity (Xu et al. 2023; Lin et al. 2024). Collectively, these studies indicate that habitat quality is declining across diverse geographic locations and landscape types, largely driven by human activities such as urban expansion, infrastructure development, mining, lake reclamation, and inadequate protection measures. These studies provide compelling evidence of human‐induced habitat quality degradation, underscoring that achieving sustainable development goals in the Anthropocene remains a long‐term and complex challenge. Additionally, existing research has explored the driving factors of habitat quality change from two primary perspectives: (1) changes in land use/land cover (LULC) and (2) the combined effects of natural environmental conditions and socioeconomic factors. LULC change not only reflects the current state of land cover but also serves as an indirect indicator of human activities to a certain extent. However, existing research has limited quantification of the contribution of LULC change to habitat quality change. Addressing this gap constitutes one of the core focuses of the present study.

Landscape fragmentation driven by human activities represents another critical perspective for evaluating habitat quality; consequently, landscape indices are widely adopted as references for assessing both habitat quality and biodiversity (Gong et al. 2019). Among these indices, landscape fragmentation and connectivity stand out as key indicators for revealing clear human impacts. For example, the Landscape Fragmentation Index shows a severe decline in the ecological connectivity and habitat quality of the Wardha River in India (Nasim et al. 2025), while urban development has notably increased landscape diversity in other regions (Maity et al. 2022). The US natural landscape has gradually grown human‐dominated over the past three decades (Theobald 2010). In Africa's Democratic Republic of the Congo, land cover proportion and disturbance indices show artificial landscapes rapidly replacing natural ones (Muteya et al. 2022). In China, landscape indices also reflect distinct regional trends. From 2000 to 2022, indices like patch density, largest patch index, landscape shape index, contagion index, Shannon's diversity index, and Shannon evenness index reveal landscape fragmentation to be complex and heterogeneous in the Yellow River Basin in Qinghai Province (Zheng et al. 2024). East China's Taihu Lake basin saw 1985–2015 urban expansion raise landscape fragmentation, sharply cutting habitat quality, especially near forestland and built‐up land (Xu et al. 2019; Pu et al. 2024). Jiangsu Province's urban land expansion drives landscape fragmentation, with clearly higher dispersion and lower cohesion (Shi et al. 2024). A 2000–2020 Poyang Lake study identifies CONTAG as the main factor driving habitat quality spatial heterogeneity (Zhang, Hao, et al. 2024). Conversely, the Yellow River's Xiaolangdi Reservoir improved habitat via reduced fragmentation and higher aggregation (Zhao et al. 2022). The application of landscape indices to evaluate the relationship between LULC change and habitat quality constitutes an important research direction. However, existing studies have primarily focused on landscape pattern characteristics of LULC change, while neglecting those associated with habitat quality change. These studies often only consider changes in habitat quality indices, overlooking equally important landscape pattern attributes. Furthermore, methods such as spatial autocorrelation analysis have not been applied to assess the landscape pattern characteristics of the dual variables of LULC and habitat quality. As a result, habitat quality changes extracted in previous research are limited to index‐based changes, failing to account for the landscape pattern characteristics and spatial interactions. To address these gaps, the present study employs landscape indices to analyze LULC and habitat quality separately and conducts bivariate spatial autocorrelation analysis on identical landscape indices to explore their spatial correlations.

Hunan Province is situated in the subtropical zone of southern China, south of the Yangtze River Basin. It features extensive distributions of evergreen broad‐leaved forests (Quan et al. 2023) and is influenced by the East Asian summer monsoon climate (Wang et al. 2021; Lin et al. 2022), collectively fostering favorable natural environmental conditions. As part of the Yangtze River Economic Belt, Hunan Province has experienced an overall trend of landscape fragmentation and habitat quality degradation, primarily driven by intensive human activities (Zheng et al. 2023; Guo, Yang, et al. 2023; He et al. 2023; Bian et al. 2024; Yang, Yang, et al. 2024). Spatially, regions with low and degraded habitat quality in Hunan Province are concentrated in urban regions along the middle and lower reaches of rivers, whereas regions with high habitat value are predominantly located in the mountainous regions of eastern and western Hunan (Hong et al. 2023). This pattern is consistent with the ecological environment quality evaluated using the remote sensing ecological index (RSEI) (Hui and Cheng 2024). Dongting Lake, a key component of Hunan Province, serves as a critical wintering habitat for waterbirds and an important ecological reserve, thereby attracting widespread research attention (Zhang et al. 2021). However, driven by socioeconomic factors, the habitat quality has also exhibited a significant degradation trend (Deng et al. 2024), especially closely associated with the terms of LULC transformation dynamics, population density, gross domestic product (GDP) density, nighttime light intensity, and LULC transformation intensity (Xuan et al. 2024). Notably, habitat quality degradation is more pronounced in the Changsha‐Zhuzhou‐Xiangtan urban agglomeration, where a unique spatial pattern of high habitat quality in the city center and low habitat quality around the city (Mi et al. 2024). Existing studies on habitat quality in Hunan Province have primarily focused on specific landscapes or geomorphic zones (e.g., wetlands and urban agglomerations), with insufficient attention paid to comprehensive assessments covering multiple environmental regions. Furthermore, while quantifying the contribution of driving factors is crucial for understanding the mechanisms underlying habitat quality changes, this remains a key challenge in current research. Additionally, gaps persist in the application of landscape indices to habitat quality assessments, which approach that could offer novel insights into understanding habitat quality dynamics. To address these research limitations, the present study employs the InVEST model and landscape indices to evaluate habitat quality changes in Hunan Province, encompassing regions with diverse environmental backgrounds and varying intensities of human activity. Specifically, this study aims to achieve four objectives: (1) analyze the overall change characteristics of LULC in Hunan Province; (2) assess spatiotemporal changes in habitat quality; (3) quantify the contribution of different LULC change types to habitat quality variations; (4) explore the spatial correlation between LULC and habitat quality based on landscape indices.

## 2 Materials and Methods

### 2.1 Study Area

**FIGURE 1:**
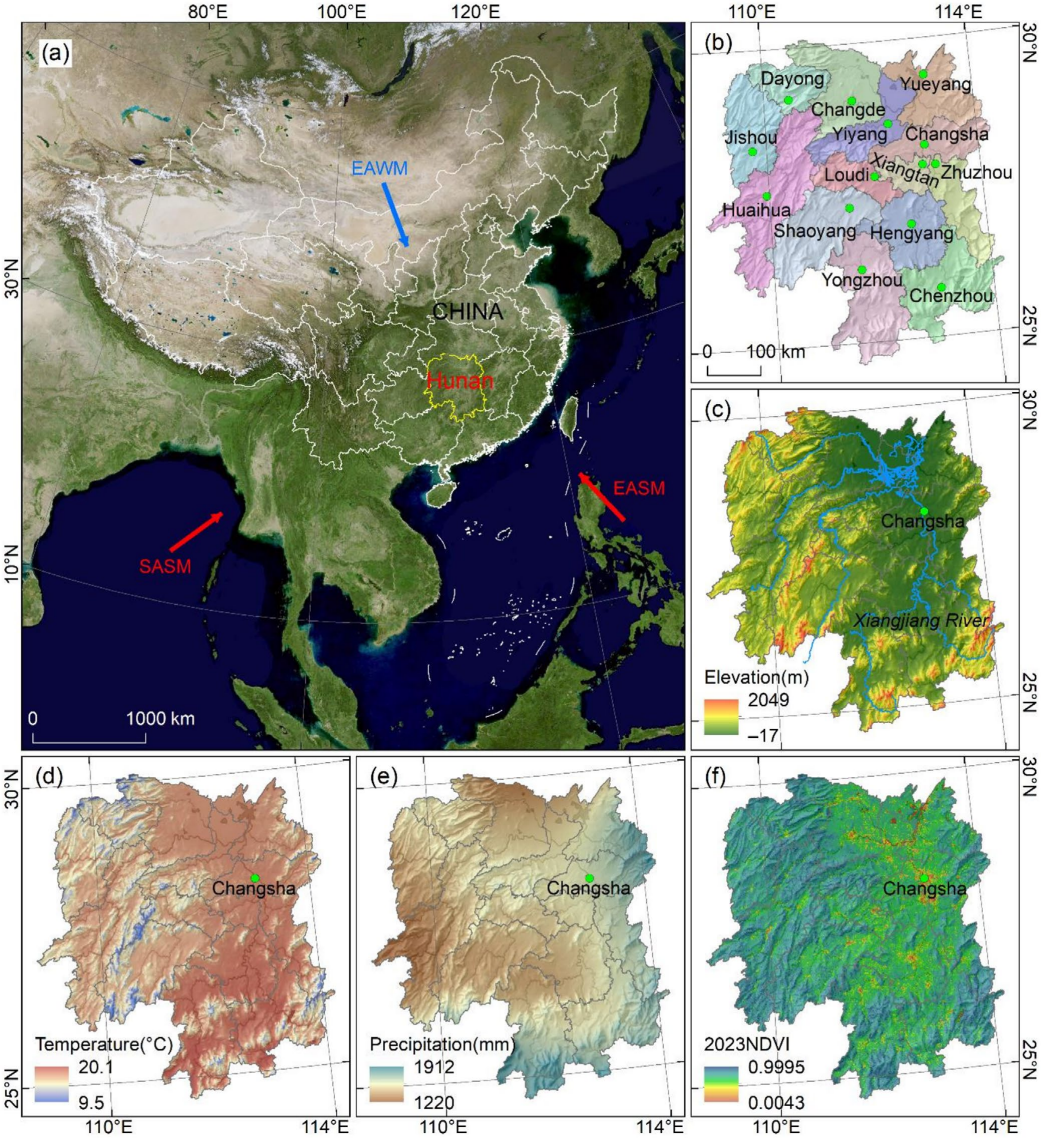
Study area. (a) Location of Hunan Province in South China; SASM, EASM, and EAWM represent South Asian Summer Monsoon, East Asian Summer Monsoon, and East Asian Winter Monsoon, respectively (Wang, Zhong, et al. 2024; Zhong et al. 2025). (b) City distribution of Hunan Province. (c) Elevation. (d, e) Annual average temperature and precipitation distribution from 1980 to 2015. (f) Normalized difference vegetation index (NDVI) in 2023.

Hunan Province (~108°45′–114°15′ E, 24°40′–30°10′ N) is located in South China and also constitutes an administrative division in central‐south China (Figure 1a). It administers 14 prefecture‐level cities, with Changsha located in the northeast part of the province serving as its capital (Figure 1b). Geomorphologically, Hunan Province lies within the southeast hills, exhibiting a topographic pattern of “higher in the south and lower in the north, higher in the west and lower in the east” (Figure 1c). The mountain regions in western Hunan form a transitional zone between the Yunnan–Guizhou Plateau and the southeast hills. Constrained by this terrain, major rivers including the Xiangjiang, Zishui, and Yuanjiang Rivers converge northward into Dongting Lake before eventually flowing into the Yangtze River (Figure 1c). Climatically, the region is dominated by a subtropical monsoon climate, shaped by the East Asian monsoon (Figure 1a) (Wang, Zhong, et al. 2024; Zhong et al. 2025). The annual average temperature ranges from ~9.5°C to 20.1°C, with annual precipitation varying between ~1200 and 2000 mm (Figure 1d,e). Both heat and water resources are primarily concentrated from March to September. Based on climatic data spanning 1959–2019, annual precipitation, as well as precipitation in spring, summer, and winter, has shown an increasing trend, whereas autumn precipitation has decreased (Lin et al. 2025). Hunan Province features relatively high vegetation coverage, with its spatial distribution closely aligned with topographic patterns. Specifically, plain and valley regions characterized by higher population densities exhibit lower vegetation coverage, while mountainous and hilly areas with smaller populations have higher vegetation coverage (Figure 1f). The vegetation in this region is dominated by evergreen broad‐leaved forests, with key taxa including species from the Fagaceae, Paulownia, and Magnoliaceae families (Liu et al. 2023).

### 2.2 Research Framework and Data

**FIGURE 2:**
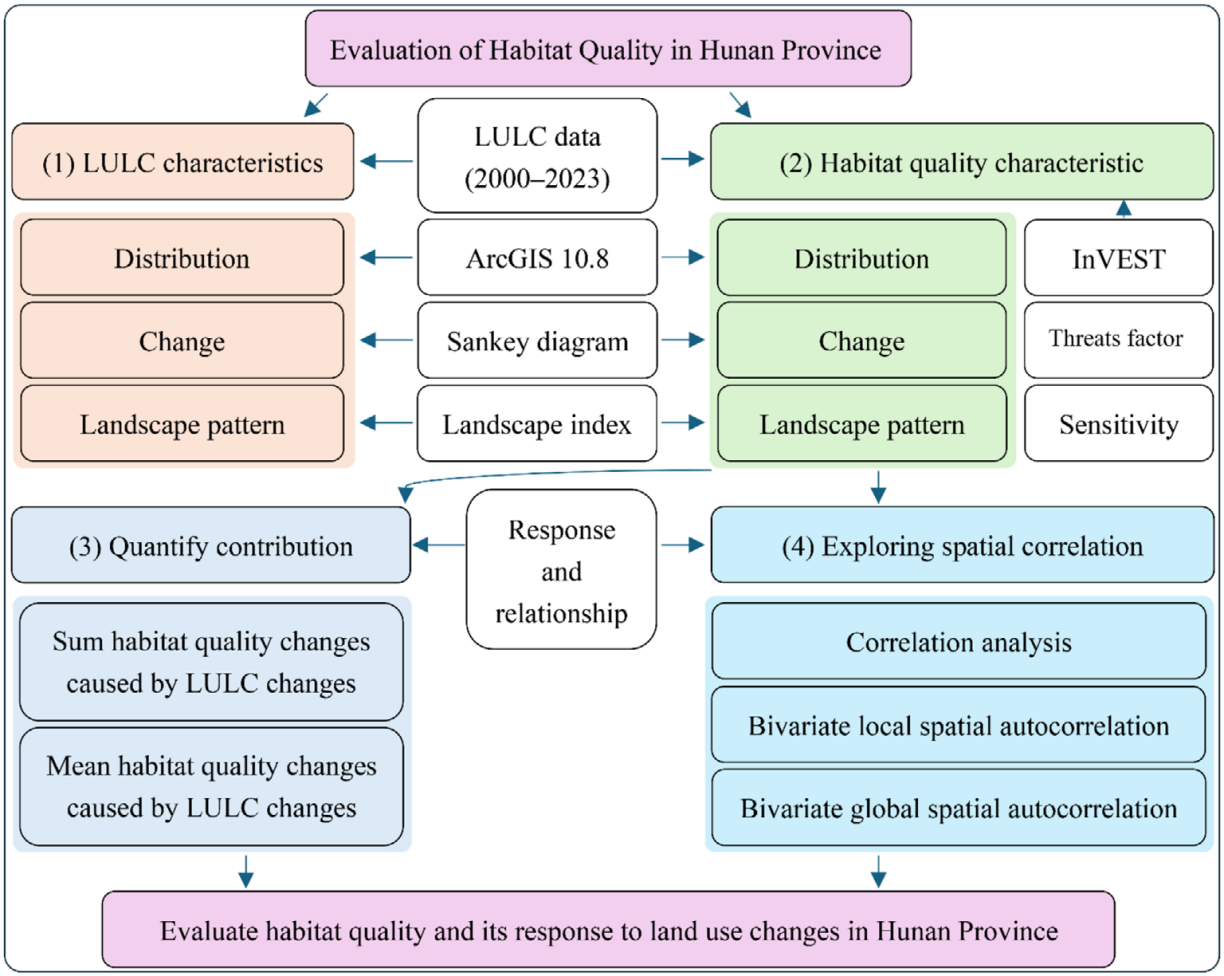
Research framework.

To address the research goal and four specific tasks of habitat quality assessment in Hunan Province, the following research framework was developed (Figure 2): (1) Using LULC data from 2000, 2005, 2010, 2015, 2020, and 2023, the spatial distribution of LULC was analyzed via the ArcGIS 10.8 platform, LULC changes were examined using a Sankey diagram, and the landscape patterns of LULC were quantified with landscape indices; (2) habitat quality was evaluated based on LULC data and the InVEST model, followed by analyses of its spatial distribution, temporal changes, and corresponding landscape patterns; (3) the contribution of LULC changes to variations in habitat quality was quantified from both cumulative sum and mean value perspectives; (4) the spatial relationship between LULC and habitat quality was explored using correlation analysis, bivariate local spatial autocorrelation, and bivariate global spatial autocorrelation.

Two types of data were utilized in this study, as detailed below:

1. *Environmental background data*. Administrative division data were obtained from relevant management authorities. Elevation data were acquired from the Shuttle Radar Topography Mission (SRTM, https://www.earthdata.nasa.gov/data/instruments/srtm). Temperature and precipitation data (1980–2015) were sourced from the National Meteorological Science Data Center (https://data.cma.cn/). Normalized Difference Vegetation Index (NDVI) data (2023) were retrieved from the Resource and Environmental Science Data Center of the Chinese Academy of Sciences (RESDC‐CAS, https://www.resdc.cn/).
2. *Data for habitat quality assessment*. Land use and land cover (LULC, 1 km) data (2000, 2005, 2010, 2015, 2020, and 2023) were obtained from the China Multi‐Period Land Use and Land Cover Remote Sensing Monitoring Dataset (CNLUCC), provided by RESDC‐CAS (https://www.resdc.cn/). Attribute data for the InVEST model were derived from existing references and official model documentation.

### 2.3 Habitat Quality Evaluation Based on InVEST Model

Biodiversity is closely related to ecosystem services, LULC changes, and biodiversity threats. Therefore, the InVEST model (v 3.14.3 Workbench, https://naturalcapitalproject.stanford.edu/software/invest) can be used to evaluate the habitat quality, degradation level, and habitat rarity of the landscape in the region by inputting LULC maps, the sensitivity of different LULC types to each threat, spatial data on the distribution and density of each threat, and the spatial coordinates of protected areas. Calculate based on the following formula (Natural Capital Project 2025; Chen et al. 2025; Hui et al. 2025; Wang, Jia, et al. 2025):

(1) $$Q_{\mathrm{xj}}=H_{j}\left[1-\left(\frac{D_{\mathrm{xj}}^{z}}{D_{\mathrm{xj}}^{z}+k^{z}}\right)\right]$$

(2) $$D_{\mathrm{xj}}=\sum_{r=1}^{R}\sum_{y=1}^{\mathrm{Yr}}\left(\omega_{r}/\sum_{r=1}^{R}\omega_{r}\right)r_{y}i_{\mathrm{rxy}}\beta_{x}S_{\mathrm{jr}}$$

In the Equation (1), *Q*_*xj*_ is the habitat quality index of the *j*‐th LULC type *x* grid unit, which is divided into low (0–0.3), lower (0.3–0.6), medium (0.6–0.75), higher (0.75–0.9), and high (0.9–1) in this study according to the evaluation results; *H*_*j*_ is the habitat suitability score for the *j*‐th LULC type, with a range of values from 0 to 1; *Z* is the scale constant, usually taken as 2.5; *k* is a half‐saturation constant, generally half of the maximum degradation degree; *D*_*xj*_ is the habitat degradation index, which represents the degree of degradation exhibited by the habitat under stress. In the Equation (2), *r* is the stress factor; *R* is the number of stress factors; *y* is a single grid in the stress factor *r* grid layer; *Y*_*r*_ is the total number of grid units of the stress factor *r*; *w*_*r*_ is the relative weight of the stress factor *r* to all habitats (Table 1); *i*_*rxy*_ represents the impact of stress factor *r* on each grid of the habitat (linear or exponential) (Table 1); *β*_*x*_ represents the level of habitat anti‐interference; *S*_*jr*_ represents the relative sensitivity of each habitat to different stress factors (Table 2).

**TABLE 1:** Threats factor weight and influence distance.

LULC	Maximum impact distance (km)	Weight	Distance decrease rate
Cultivated land	1	0.6	Linear
Urban land	10	0.8	Exponential
Rural residential areas	5	0.6	Exponential
Other built‐up land	2	0.7	Exponential
Unused land	1	0.5	Linear

**TABLE 2:** Habitat suitability of different LULC types and sensitivity to stress factors.

LULC	Habitat suitability	Threats factor				
		Cultivated land	Urban land	Rural residential areas	Other built‐up land	Unused land
Paddy field	0.7	0	0.9	0.7	0.8	0.4
Dry land	0.5	0	0.8	0.6	0.7	0.3
Closed forestland	1	0.7	0.9	0.8	0.8	0.5
Shrubs forestland	0.9	0.6	0.8	0.6	0.7	0.4
Sparse forestland	0.8	0.7	0.8	0.7	0.8	0.5
Other forestland	0.7	0.7	0.8	0.7	0.8	0.4
High coverage grassland	0.8	0.6	0.7	0.7	0.7	0.7
Medium coverage grassland	0.7	0.6	0.7	0.7	0.7	0.7
Low coverage grassland	0.5	0.6	0.7	0.7	0.7	0.7
Rivers and canals	0.7	0.4	0.7	0.6	0.7	0.4
Lake	0.8	0.4	0.8	0.6	0.7	0.4
Reservoir pits and ponds	0.7	0.4	0.8	0.6	0.7	0.4
Mudflat	0.6	0.4	0.6	0.5	0.6	0.3
Beach land	0.6	0.4	0.6	0.5	0.6	0.3
Urban land	0	0	0	0	0	0
Rural residential areas	0	0	0	0	0	0
Other built‐up land	0	0	0	0	0	0
Marshland	0.6	0.4	0.6	0.5	0.6	0.3
Bare land	0.2	0.3	0.5	0.4	0.5	0
Bare rocky land	0.1	0.3	0.5	0.4	0.5	0

Habitat quality evaluation based on InVEST model requires four types of data, including LULC, threat factor data and tables (Table 1), sensitivity table (Table 2), and half‐saturation constant (Wang, Jia, et al. 2025).

### 2.4 Landscape Indices Based on Fragstats

Landscape indices are defined as a concise quantitative indicator that highly condenses landscape pattern information, while reflecting specific aspects of landscape structural composition and spatial configuration characteristics. It serves as a spatial analysis approach well‐suited for quantitatively describing the correlation between landscape patterns and ecological processes. In this study, landscape indices were calculated using Fragstats (v 4.3.833b, https://www.fragstats.org), with the moving window method selected for the computation process.

Largest patch index (LPI), edge density (ED), fractal dimension index (FRAC), patch density (PD), patch cohesion index (COHESION), and Shannon's diversity index (SHDI) were selected for this study. The specific calculations are as follows (Liu et al. 2022; Liu, Chang, et al. 2024; Liu, Liu, et al. 2024; Zhang, Wan, et al. 2024; Wang, Liu, et al. 2025):

(3) $$\mathrm{LPI}=\frac{\max\left(a_{\mathrm{ij}}\right)}{A}\times 100\%$$

(4) $$\mathrm{ED}=\frac{E}{A}\times 10000$$

(5) $$\text{FRAC}=2\times \frac{\ln\left(0.25\times p_{\mathrm{ij}}\right)}{\ln a_{\mathrm{ij}}}$$

(6) $$\mathrm{PD}=\frac{N}{A}\times 10000\times 100\%$$

(7) $$\text{COHESION}=\left[1-\frac{\sum_{i=1}^{m}\sum_{j=1}^{n}p_{\mathrm{ij}}}{\sum_{i=1}^{m}\sum_{j=1}^{n}p_{\mathrm{ij}}\sqrt{a_{\mathrm{ij}}}}\right]/\left[1-\frac{1}{\sqrt{Z}}\right]\times 100\%$$

(8) $$\text{SHDI}=-\sum_{i=1}^{m}\left(p_{i}\times \ln p_{i}\right)$$

In the Equation (3), *LPI* is Largest Patch Index, it ranged from 0 to 100, and high value represents that a certain landscape type has a significant influence and advantageous position in the ecosystem; *a*_*ij*_ is the area (m^2^) of patch *ij*; *A* is the total landscape area (m^2^). In the Equation (4), *ED* is Edge Density; it ≥ 0 and high value represents that the edge length per unit area in the landscape is longer and the degree of landscape fragmentation is higher; *E* is the total length (m) of edge in the landscape; *A* is the total landscape area (m^2^). In the Equation (5), *FRAC* is Fractal Dimension Index, it ranged from 1 to 2 representing simplicity to complexity; *p*_*ij*_ is the perimeter (m) of patch *ij*; *a*_*ij*_ is the area (m^2^) of patch *ij*. In the Equation (6), *PD* is Patch Density; it ≥ 0 and high value represents a complex landscape with high heterogeneity and fragmentation; *N* is the total number of patches in the landscape; *A* is the total landscape area (m^2^). In the Equation (7), *COHESION* is Patch Cohesion Index, it ranged from 0 to 100 and higher value represents better connectivity between patches and the stronger the connectivity of the landscape; *p*_*ij*_ is the perimeter of patch *ij* in terms of number of cell surfaces; *a*_*ij*_ is the area of patch *ij* in terms of number of cells; *Z* is the total number of cells in the landscape. In the Equation (8), *SHDI* is Shannon's Diversity Index; it ≥ 0 and without limit, and larger *SHDI* value represents the higher the diversity of species in the community; *P*_*i*_ is the proportion of the landscape occupied by patch type *i*.

### 2.5 Response Analysis of Habitat Quality to LULC

Calculate the increase or decrease in regional habitat quality caused by a certain land use change to measure the response of habitat quality to land use change (Wang, Liu, Xiang, et al. 2023). This study calculates the changes in habitat quality using the following formula:

(9) $$∆\mathrm{LHQ}={\mathrm{LHQ}}_{t+1}-{\mathrm{LHQ}}_{t}$$

In the Equation (9), *∆LHQ* is the change value of habitat quality response to LULC changes; we calculated the sum and mean values in this study: *LHQ*_*t*+1_ and *LHQ*_*t*_ are the final and initial habitat quality index for a certain type of LULC, respectively.

### 2.6 Spatial Autocorrelation Analysis

Spatial autocorrelation refers to the correlation and degree between the observed values at a certain location in space and those at adjacent locations. It is used to verify whether the attribute value of a certain element is significantly correlated with the attribute value of its adjacent position. The popular software is GeoDa (v1.22, https://geodacenter.github.io/). The quantitative methods for spatial autocorrelation include global spatial autocorrelation indicators (such as Moran's *I*) and local spatial autocorrelation indicators (such as LISA). This study used bivariate spatial autocorrelation, and the specific calculations are as follows (Liu, Chang, et al. 2024; Liu, Liu, et al. 2024; Ai et al. 2024):

(10) $$I=\frac{\sum_{i=1}^{n}\sum_{j=1}^{n}W_{\mathrm{ij}}\left(x_{i}-\bar{x}\right)\left(y_{i}-\bar{y}\right)}{S^{2}\sum_{i=1}^{n}\sum_{j=1}^{n}W_{\mathrm{ij}}}$$

(11) $${\text{LISA}}_{i}=z_{i}\times \sum_{j=1}^{n}w_{\mathrm{ij}}\times z_{j}$$

In the Equation (10), *I* is the bivariate global spatial autocorrelation index; *n* is the total number of space units; *W*_*ij*_ is the spatial weight matrix; *x*_*i*_ and *y*_*j*_ are the observed values; and *S* ^*2*^ is the variance of all samples. In the Equation (11), *LISA*_*i*_ is the Local Indicators of Spatial Association, and the clustering mode can be divided into High–High (H–H) aggregation, Low–Low (L–L) aggregation, Low–High (L–H) aggregation, and High–Low (H–L) aggregation; *z*_*i*_ and *z*_*j*_ are the normalized values of the variances of the observations.

## 3 Results

### 3.1 Spatial–Temporal Characteristics of LULC

#### 3.1.1 LULC Distribution Characteristics

**FIGURE 3:**
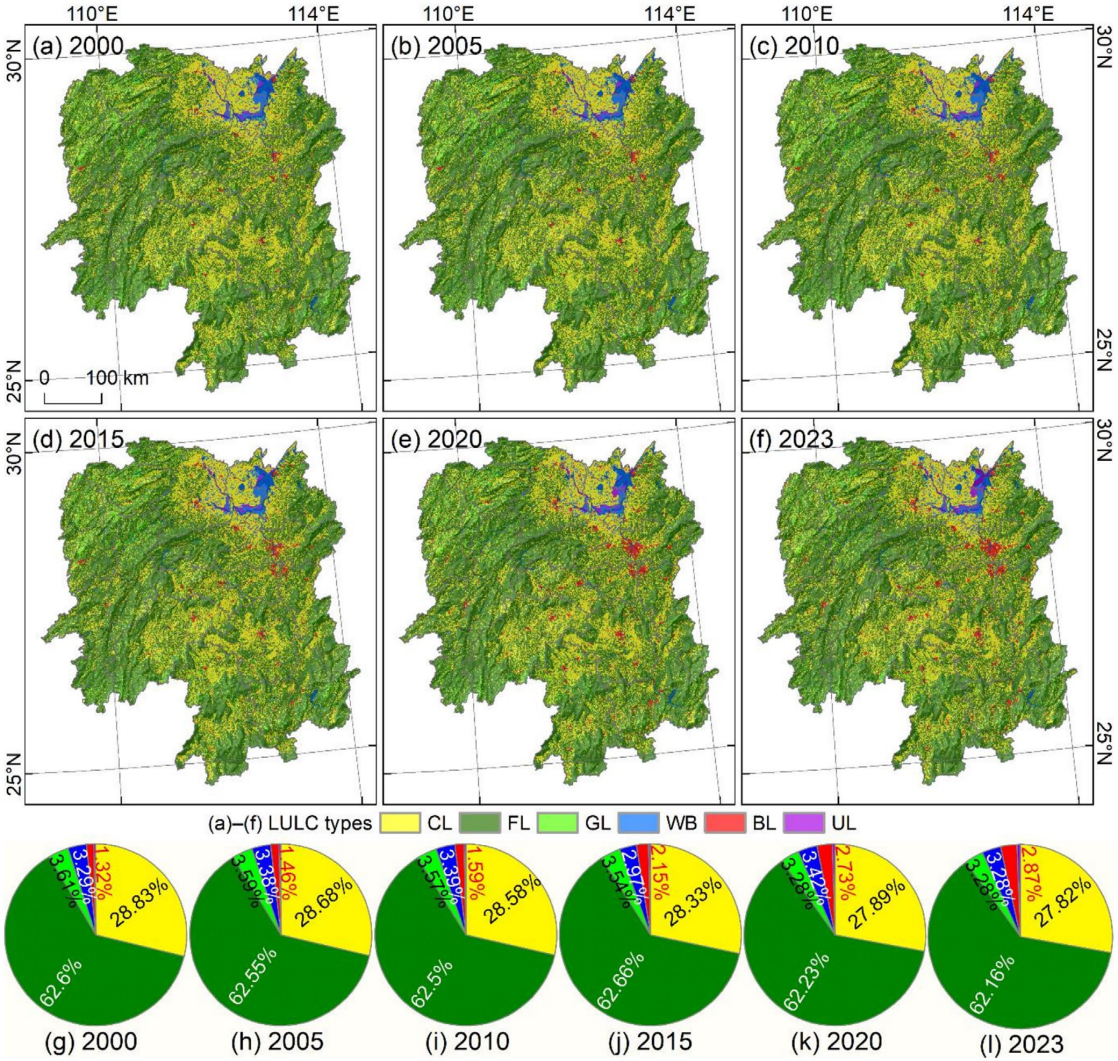
LULC distribution in Fujian Province. (a–f) LULC from 2000 to 2023; CL, FL, GL, WB, BL, and UL represent cultivated land, forestland, grassland, water body, built‐up land, and unused land, respectively. (g–l) Area proportion of LULC from 2000 to 2023.

The LULC structure of Hunan Province is dominated by forestland, which accounts for 62.16%–62.6% of the total area and is primarily distributed in the mountainous regions of western, southern, and eastern Hunan (Figure 3). This is followed by cultivated land, which constitutes 27.82%–28.83% of the total area and is mainly distributed within the middle and lower reaches of rivers in the central and northern plains, as well as in the vicinity of lakes. Grassland is scattered across the mountainous regions of northwestern Hunan, while water bodies are predominantly concentrated in Dongting Lake in northern Hunan, and built‐up land is sporadically distributed throughout the plains of central and northern Hunan.

Spatial distribution and area changes further indicate a steady expansion of built‐up land (Figure 3). Although built‐up land was only scattered across multiple locations in 2000, accounting for 1.32% of the total area (Figure 3a–g), it had exhibited a distinct expansion trend increasing to 2.87% in 2023 (Figure 3f–l). Notably, the built‐up land in Changsha–Zhuzhou–Xiangtan urban agglomeration showed a clear clustered distribution pattern (Figure 3f).

#### 3.1.2 Landscape Indices of LULC

**FIGURE 4:**
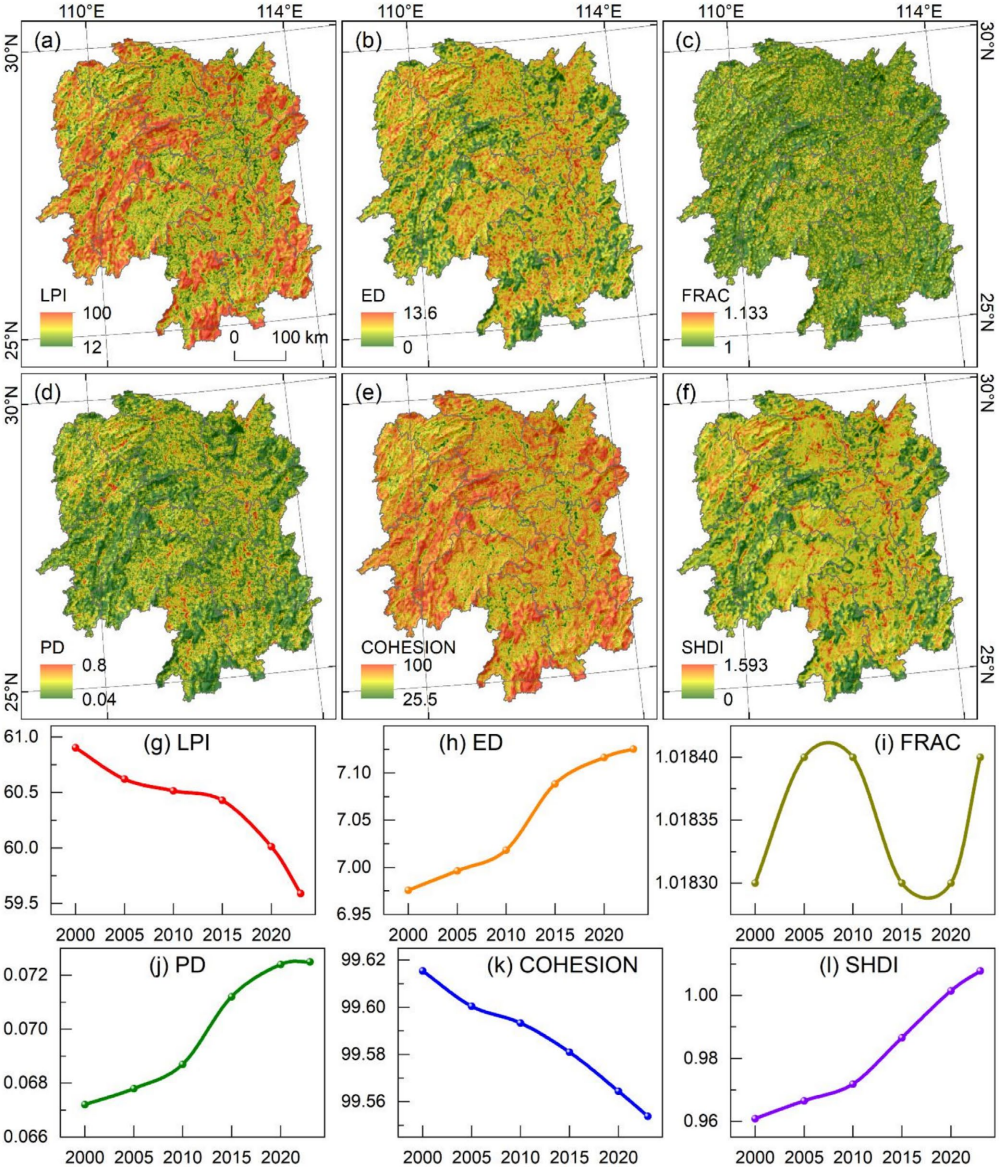
Landscape index of LULC in Hunan Province. (a–f) Landscape indices distribution in 2023. (g–l) Landscape indices change from 2000 to 2023.

The landscape indices distribution of LULC exhibits significant differences (Figure 4a–f). Due to the similar spatial patterns of the same index across different years, only the data for 2023 are presented herein. The largest patch index (LPI) is higher in the mountainous regions of western, southern, and eastern Hunan, while lower values are observed in valley regions (Figure 4a). The edge density (ED) shows a roughly opposite distribution to LPI, is relatively high in river valleys and lower in mountainous areas in the western, southern, and eastern Hunan (Figure 4b). The fractal dimension index (FRAC) is generally low, with no significant spatial differences (Figure 4c). The patch density (PD) is also generally low, with relatively higher values only in valley regions, presenting a strip‐like distribution pattern (Figure 4d). The patch cohesion index (COHESION) is generally higher than PD, and it is only relatively low in river valleys, also showing a strip‐shaped pattern (Figure 4e). The Shannon diversity index (SHDI) exhibits a distribution similar to that of ED, with lower values in the western, southern, and eastern mountainous areas and relatively higher values in valley regions (Figure 4f).

The landscape indices of LULC have undergone continuous changes over the study period (Figure 4g–l). A continuous decrease in LPI indicates a reduction in the size of dominant patches and a deterioration in ecological connectivity (Figure 4g). The continuous increase in ED suggests heightened landscape fragmentation and a decline in ecosystem stability (Figure 4h). The FRAC fluctuates repeatedly, implying that patch shapes remain relatively simple and stable, while ecosystems are relatively simplistic with low diversity (Figure 4i). The steady increase in PD indicates an increase in landscape fragmentation and heterogeneity (Figure 4j). A continuous decrease in COHESION reflects a deterioration in the spatial connectivity of patches, accompanied by reduced landscape integrity and stability of the landscape (Figure 4k). The consistent increase in SHDI indicates enhanced ecosystem complexity and stability (Figure 4l).

#### 3.1.3 LULC Transfer Characteristics

**FIGURE 5:**
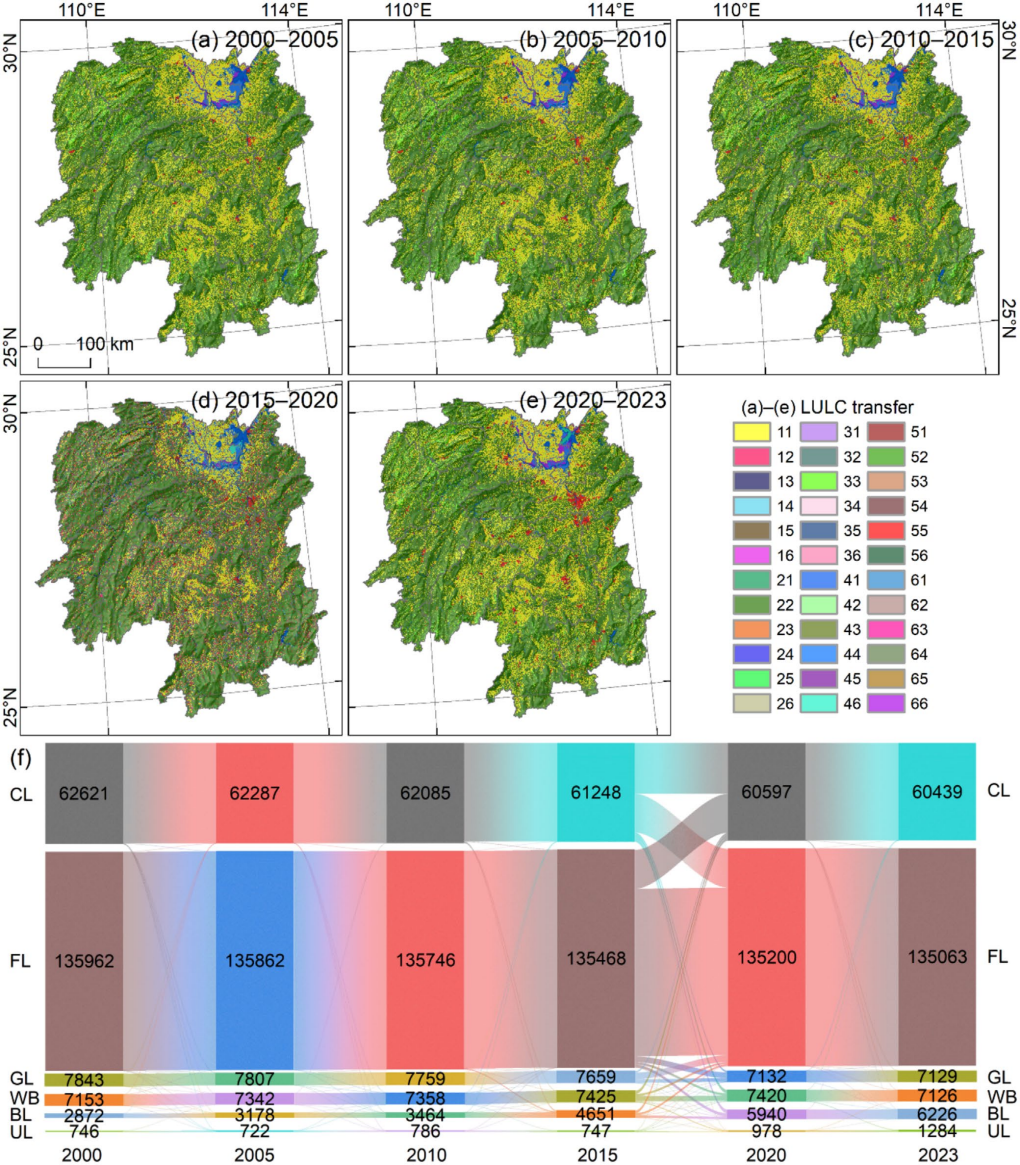
LULC transfer in Hunan Province from 2000 to 2023. (a–e) LULC transfer distribution; in the legend, two‐digit codes represent the LULC types before and after the transfer. (f) LULC transfer area from 2000 to 2023.

The transfer characteristics of LULC were primarily spatially invariant, particularly in the distribution of forestland, and the most intense transfer occurred during the 2015–2020 period (Figure 5). From 2000 to 2005, among the transfers between different land types, the conversion of cultivated land to water bodies (131 km^2^), cultivated land to built‐up land (153 km^2^), and forestland to built‐up land were relatively common (151 km^2^) (Figure 5a,f). Between 2005 and 2010, the dominant transfers included cultivated land to built‐up land (141 km^2^), and forestland to built‐up land (139 km^2^) (Figure 5b,f). The magnitude of LULC transfer from 2010 to 2015 was significantly higher than that during the 2000–2010 period; key transfers during this phase included cultivated land to forestland (237 km^2^), cultivated land to built‐up land (618 km^2^), and forestland to built‐up land (572 km^2^) (Figure 5c,f). The 2015–2020 period represented the most intense LULC transfer phase throughout the study period. Specifically, relatively frequent transfers were observed between cultivated land and forestland (over 24,000 km^2^), cultivated land and grassland (over 1000 km^2^), cultivated land and water bodies (over 2000 km^2^), cultivated land and built‐up land (over 2000 km^2^), forestland and grassland (over 3600 km^2^), forestland and water bodies (over 1000 km^2^), and forestland and built‐up land (over 1000 km^2^) (Figure 5d,f). From 2020 to 2023, a relatively large amount of cultivated land (149 km^2^) and forestland (130 km^2^) was converted to built‐up land (Figure 5e,f).

### 3.2 Spatial–Temporal Characteristics of Habitat Quality

#### 3.2.1 Habitat Quality Distribution Characteristics

**FIGURE 6:**
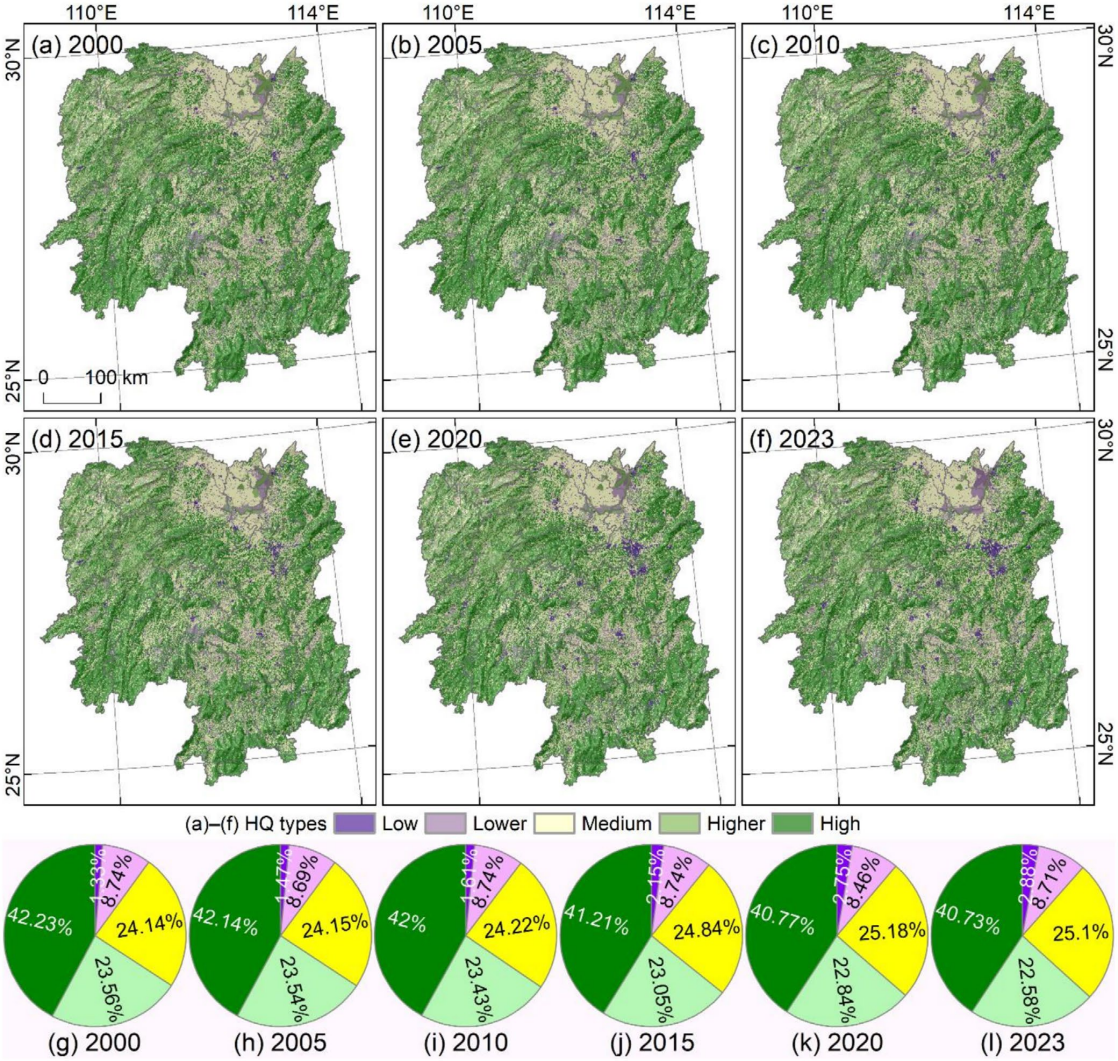
Habitat quality distribution in Hunan Province. (a–d) Habitat quality distribution from 2000 to 2023. (g–l) Area proportion of habitat quality.

Habitat quality in Hunan Province is predominantly of high quality, accounting for 40.73%–42.23% of the total area, and it is mainly distributed in the mountainous regions of southwestern, southern, and eastern Hunan (Figure 6). Areas of higher‐quality (22.58%–23.56%) and medium‐quality (24.14%–25.1%) habitat also occupy relatively large proportions. Specifically, higher‐quality habitat is primarily distributed in the vicinity of high‐quality regions, while medium‐quality habitat is clearly concentrated around lakes in northern Hunan. Low‐quality habitat is mainly distributed in the Changsha–Zhuzhou–Xiangtan urban agglomeration and river valley regions.

Spatial distribution and area changes further reveal the fragmentation of high‐quality regions and the steady expansion of low‐quality regions (Figure 6). The low‐quality regions have expanded significantly in the Changsha–Zhuzhou–Xiangtan urban agglomeration from scattered distribution in 2000 to the formation of clustered patches by 2023. Between 2000 and 2023, the low‐quality area exhibited the most significant increase (3368 km^2^, 1.55%), whereas the high‐quality habitat area showed the most substantial decrease (3214 km^2^, −1.49%). These trends collectively indicate a downward trajectory in overall habitat quality across Hunan Province.

#### 3.2.2 Landscape Indices of Habitat Quality

**FIGURE 7:**
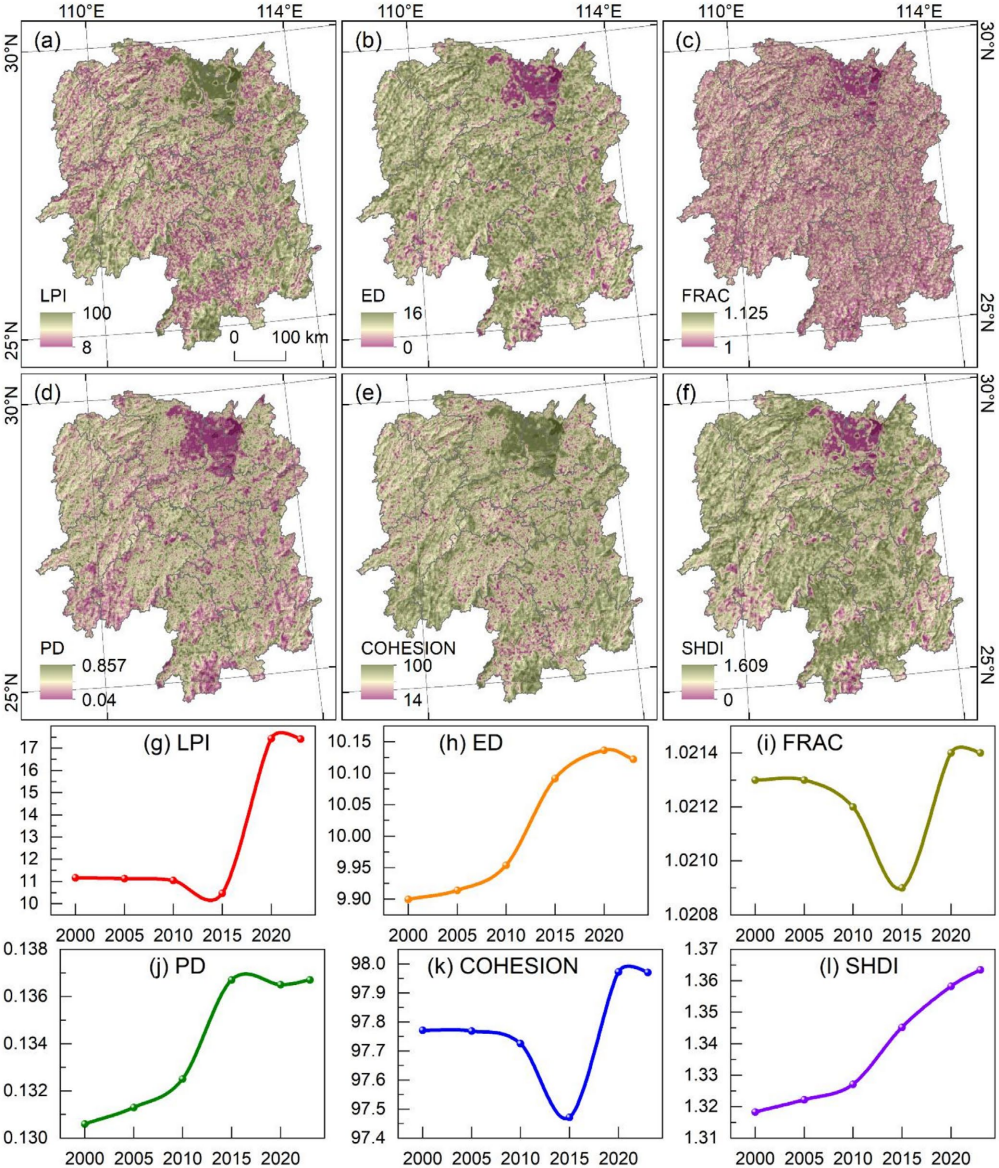
Landscape Index of habitat quality in Hunan Province. (a–f) Landscape indices distribution in 2023. (g–l) Landscape indices change from 2000 to 2023.

The spatial distribution of landscape indices for habitat quality exhibits significant variations (Figure 7a–f). Only the 2023 data are presented herein due to the similar spatial patterns of the same indices across different years. The LPI is higher in northern Hunan (Dongting Lake region) and lower in western and central Hunan (Figure 7a). The ED shows a roughly opposite distribution to LPI; it is relatively high in western and central Hunan, while lower in northern (Dongting Lake region) (Figure 7b). The FRAC is generally low, with no significant spatial differences (Figure 7c). The PD is also generally low, particularly in northern Hunan (Dongting Lake), and only relatively high in valley regions, presenting a strip‐like distribution pattern (Figure 7d). The COHESION is generally higher than PD, with notably higher values in northern Hunan (Dongting Lake), and it is only relatively low in valley regions, also showing a strip‐shaped pattern (Figure 7e). The SHDI exhibits a distribution similar to that of ED, with lower values in northern Hunan (Dongting Lake region) and relatively higher values in western and central Hunan (Figure 7f).

Notable differences are observed in the landscape indices temporal changes of habitat quality (Figure 7g–l). The LPI increased sharply between 2015 and 2020, indicating an expansion in the size of dominant habitat patches and improved connectivity of habitat quality during this period (Figure 7g). The ED increased significantly in the early stages but began to decline after 2020; this trend suggests an initial increase in habitat quality fragmentation and a decrease in habitat stability, followed by an improvement in 2020 and beyond (Figure 7h). The FRAC decreased sharply from 2010 to 2020 and then increased rapidly, reflecting significant fluctuations in habitat quality (Figure 7i). The continuous increase in PD indicates a gradual rise in habitat quality fragmentation and landscape heterogeneity (Figure 7j). The changes in COHESION are similar to those of FRAC, suggesting significant fluctuations in the spatial connectivity, integrity, and stability of habitat quality, particularly during the 2010–2020 period (Figure 7k). The SHDI increased consistently over the study period, indicating enhanced complexity and stability of habitat quality (Figure 7l).

#### 3.2.3 Habitat Quality Transfer Characteristics

**FIGURE 8:**
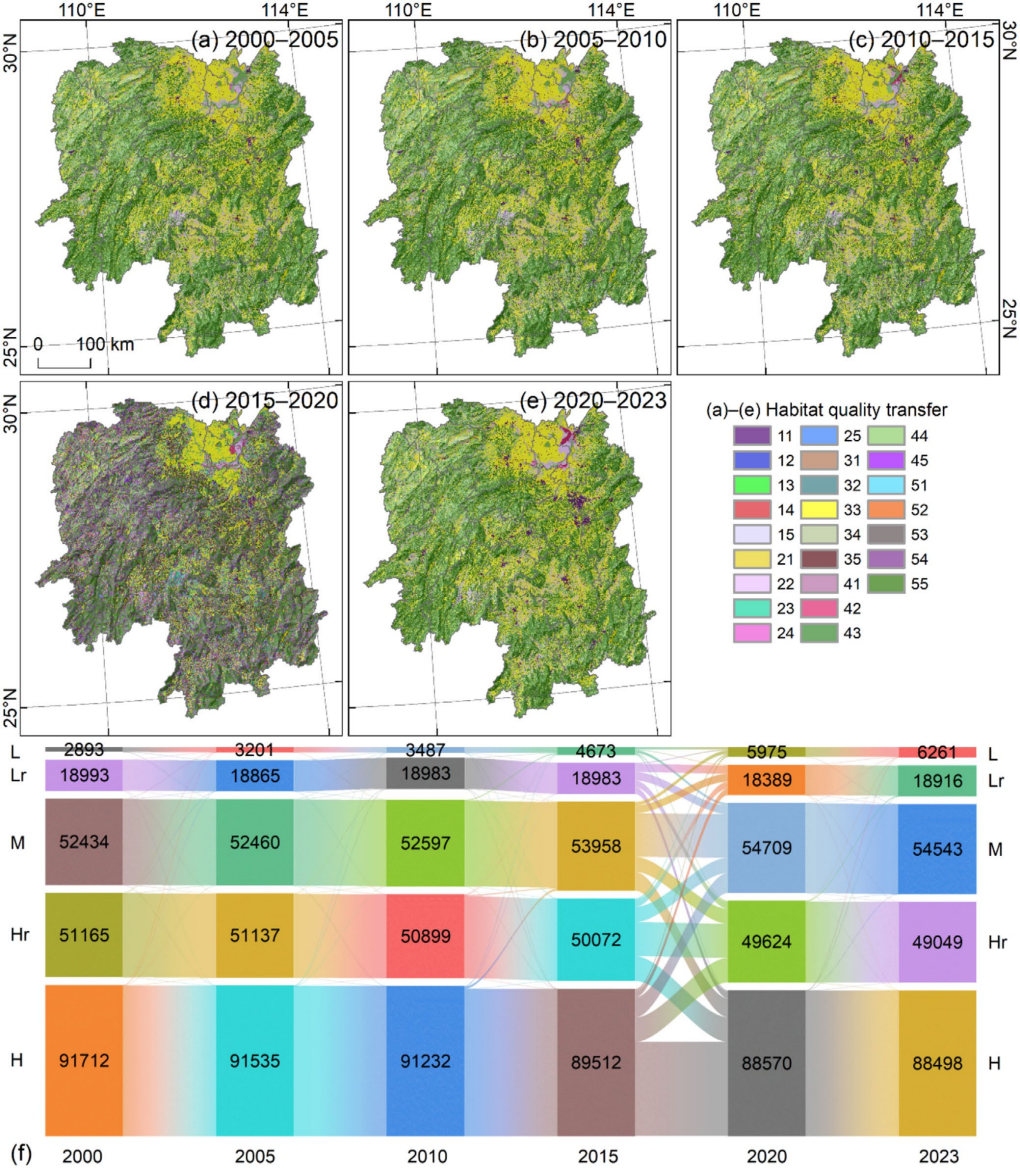
Habitat quality in Hunan Province from 2000 to 2023. (a–e) Habitat quality transfer distribution; in the legend, two codes represent the habitat quality types before and after the transfer. (f) Habitat quality transfer area from 2000 to 2023.

The transfer characteristics of habitat quality were primarily spatially invariant, particularly in the distribution of high‐quality habitat. Notably, habitat quality transfers were most intense during the 2015–2020 period, exhibiting an overall trend of habitat quality deterioration (Figure 8). Transfer between different habitat quality grades showed distinct patterns across periods. From 2000 to 2005, relatively common transfers included the conversion of lower‐quality to higher‐quality habitat (112 km^2^), medium‐quality to low‐quality (127 km^2^), and high‐quality to medium‐quality habitat (174 km^2^) (Figure 8a,f). Between 2005 and 2010, the dominant transfers were medium‐quality to low‐quality (114 km^2^), higher‐quality to lower‐quality (139 km^2^), and high‐quality to medium‐quality habitat (216 km^2^) (Figure 8b,f). The magnitude of habitat quality transfer from 2010 to 2015 was significantly higher than that during the 2000–2010 period; key transfers in this phase included medium‐quality to low‐quality habitat (507 km^2^), higher‐quality to medium‐quality habitat (536 km^2^), and high‐quality to medium‐quality habitat (1368 km^2^) (Figure 8c,f). The 2015–2020 period represented the most intense phase of habitat quality transfers throughout the study period. Specifically, frequent mutual transfers were observed between low‐quality and medium‐quality habitat (over 1600 km^2^), low‐quality and medium‐quality habitat (over 4700 km^2^), medium‐quality and high‐quality habitat (over 9100 km^2^), medium‐quality and high‐quality habitat (over 11,000 km^2^), and higher‐quality and high‐quality habitat (over 14,800 km^2^) (Figure 8d,f). From 2020 to 2023, a relatively large area of higher‐quality habitat was converted to lower‐quality habitat (520 km^2^) (Figure 8e,f). Overall, the transfer dynamics of habitat quality revealed a consistent trend that relatively high habitat quality transfers to relatively low habitat quality. This pattern indicates a gradual deterioration of habitat quality in Hunan Province from 2000 to 2023.

### 3.3 Response and Relationship of Habitat Quality to LULC

#### 3.3.1 Response of Habitat Quality to LULC Changes

**FIGURE 9:**
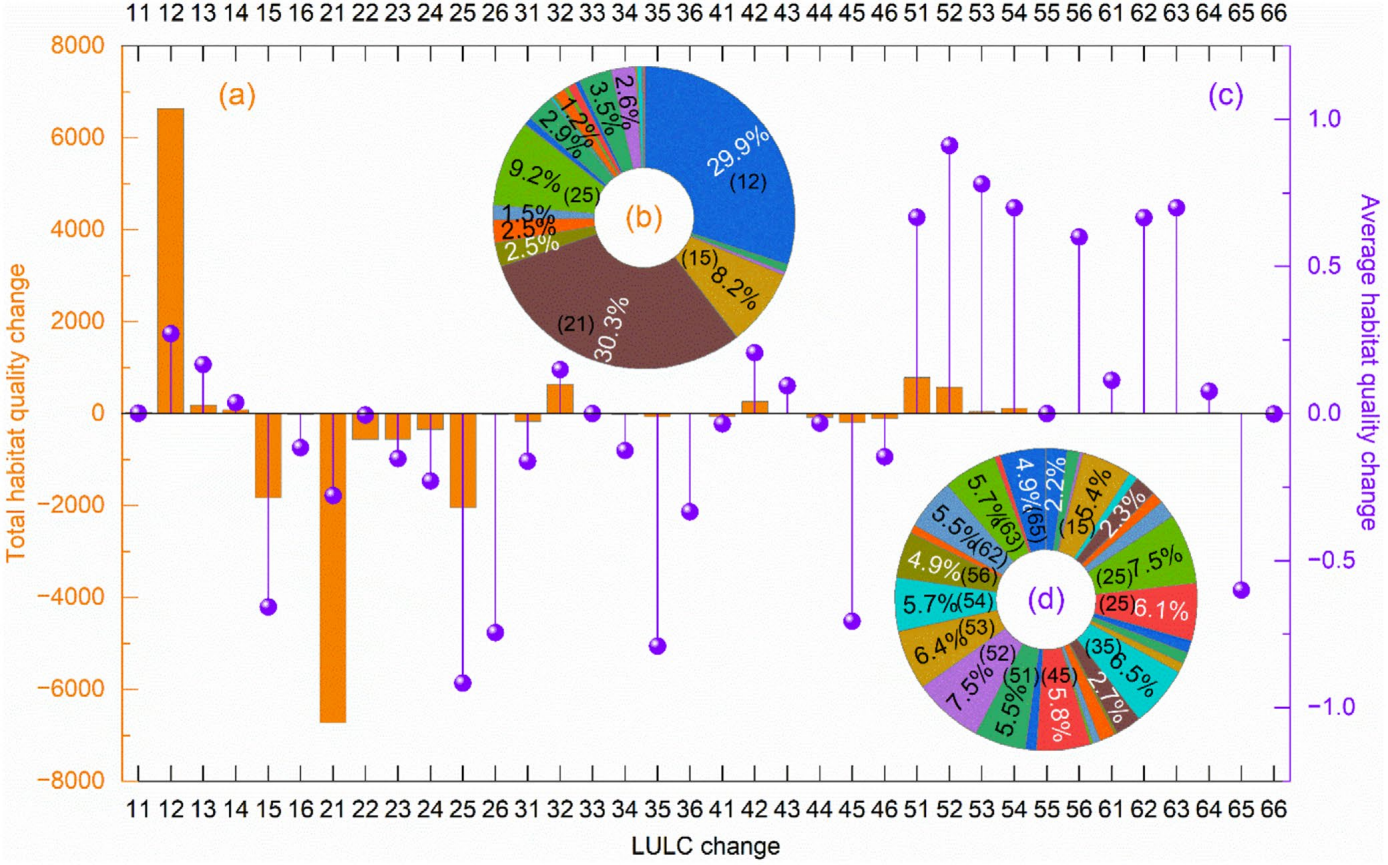
Response of habitat quality to LULC changes in Hunan Province from 2000 to 2023. (a, b) Total habitat quality changes and its proportion caused by LULC changes, respectively. (c, d) Average of habitat quality changes and its proportion caused by LULC changes, respectively. The two codes of LULC changes represent the LULC types before and after the transfer.

The habitat quality changes in Hunan Province from 2000 to 2023 were primarily driven by the mutual transfer of cultivated land and forestland, as well as the mutual transfer of built‐up land and other land (Figure 9). For total habitat quality changes, the most significant contributions came from the transfer of cultivated land to forestland (6639.58, 29.86%) and forestland to cultivated land (−6728.92, 30.26%), which exerted positive and negative effects, respectively (Figure 9a,b). In addition, conversions of cultivated land to built‐up land (−1830.44, 8.23%) and forestland to built‐up land (−2051.27, 9.23%) also made notable negative contributions. Overall, the total negative contribution to habitat quality changes (−12,833.07, 57.71%) exceeded the total positive contribution (9402.43, 42.29%), indicating an overall downward trend in habitat quality.

For mean habitat quality changes, the most prominent driver was the mutual transfer between built‐up land and other land types, with multiple conversion processes contributing over 0.5 (Figure 9c,d). Specifically, conversions from other land types to built‐up land primarily exerted negative contributions, including cultivated land to built‐up land (−0.657, 5.4%), forestland to built‐up land (−0.916, 7.53%), forestland to unused land (−0.74, 6.12%), grassland to built‐up land (−0.79, 6.49%), water bodies to built‐up land (−0.71, 5.8%), and unused land to built‐up land (−0.6, 4.93%). In contrast, conversions from built‐up land to other land types mainly exerted positive contributions, such as built‐up land to cultivated land (0.67, 5.48%), forestland (0.91, 7.48%), grassland (0.78, 6.41%), water bodies (0.7, 5.75%), unused land (0.6, 4.93%), forestland (0.67, 5.48%), and grassland (0.7, 5.75%).

#### 3.3.2 Relationship of Habitat Quality to LULC Based on Landscape Index

**FIGURE 10:**
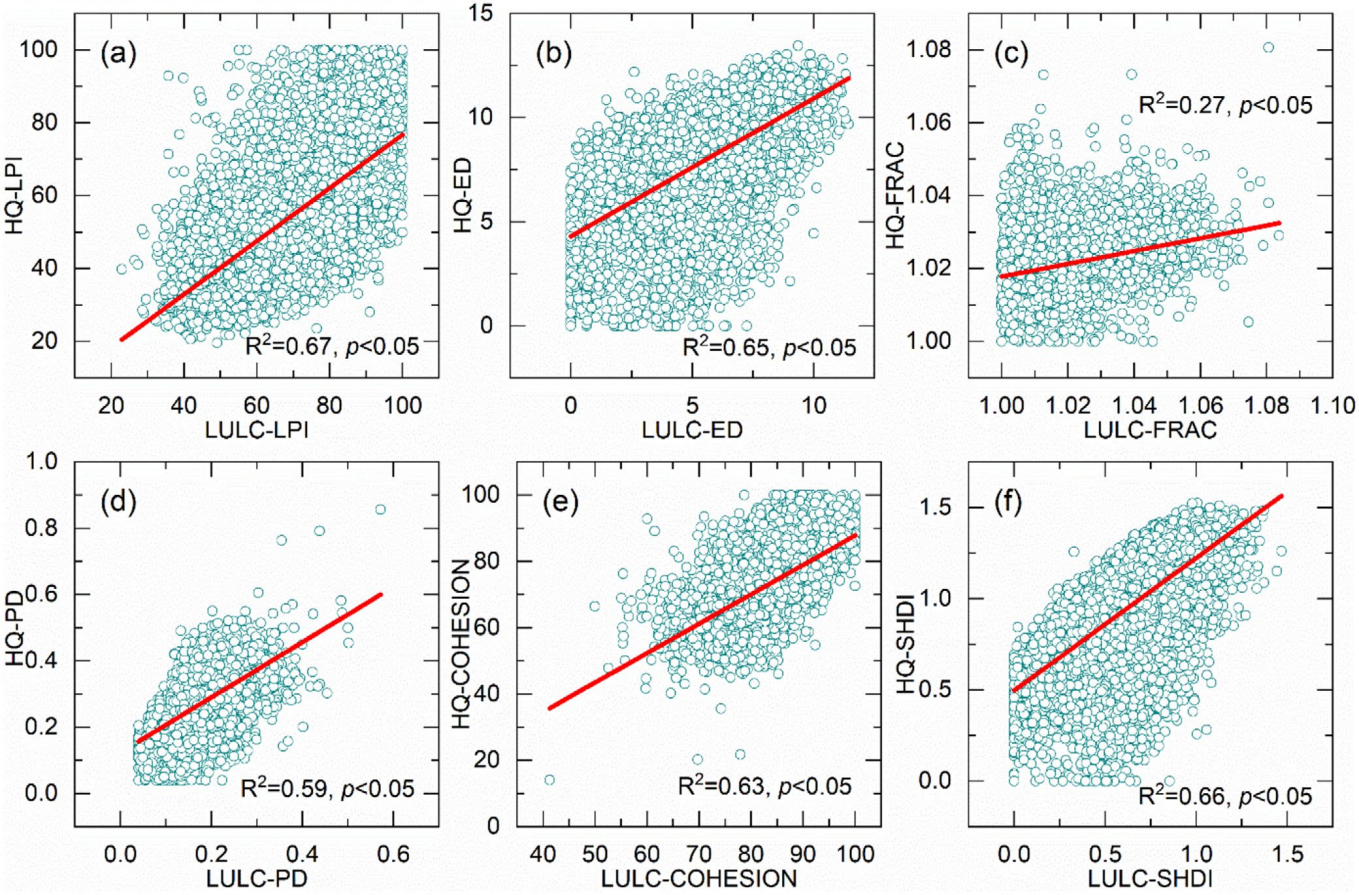
Landscape index correlation between LULC and habitat quality in Hunan Province in 2023.

A strong correlation was observed between the landscape indices of LULC and habitat quality in Hunan Province in 2023 (Figure 10). Specifically, the correlation coefficients between LULC and habitat quality in LP, ED, PD, COHESION, SHDI were close to or exceed 0.6. These results indicate a robust spatial relationship between the landscape indices' spatial distribution of LULC and habitat quality in Hunan Province, supporting the need for further spatial autocorrelation analysis.

**FIGURE 11:**
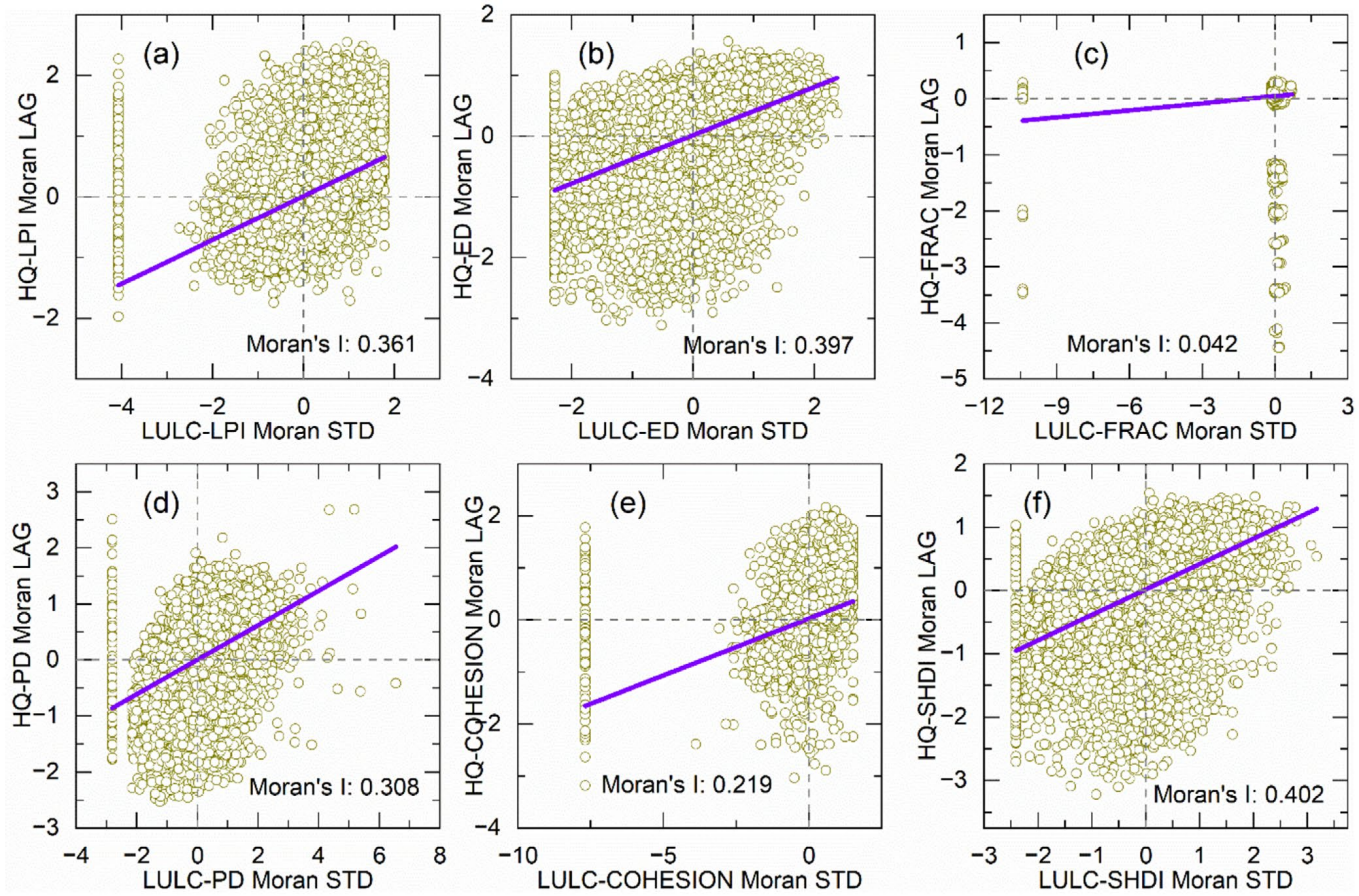
Bivariate global spatial autocorrelation of landscape index for LULC and habitat quality in Hunan Province in 2023.

The bivariate global spatial autocorrelation of the landscape indices between LULC and habitat quality in Hunan Province in 2023 revealed a strong positive spatial correlation (Figure 11, Table 3). Among the indices, Moran's *I* values for the bivariate global spatial autocorrelation of the landscape indices of LULC and habitat quality in LPI, ED, PD, and SHDI exceeded 0.3, and COHESION exceeded 0.2. These values confirm a strong spatial correlation between the spatial distribution of landscape indices for LULC and habitat quality in Hunan Province, characterized by similar spatial clustering patterns. Reliability and validity analysis of the bivariate global spatial autocorrelation showed that all landscape indices had *p* values < 0.001 and *Z*‐scores > 2.58, further verifying a statistically significant positive spatial correlation between the landscape index of LULC and habitat quality (Table 3). Overall, the bivariate global spatial autocorrelation results indicate that higher values of LULC landscape indices correspond to higher values of habitat quality related landscape indices, particularly for LPI, ED, PD, COHESION, and SHDI.

**TABLE 3:** Reliability and validity of bivariate spatial autocorrelation of landscape index.

	*I*	*p*	SD	*Z*‐score
LPI	0.361	0.001	0.0047	75.9755
ED	0.397	0.001	0.0046	85.4666
FRAC	0.0418	0.001	0.0054	7.7463
PD	0.3081	0.001	0.0045	67.92
COHESION	0.2193	0.001	0.0049	44.74
SHDI	0.4018	0.001	0.0046	87.2939

**FIGURE 12:**
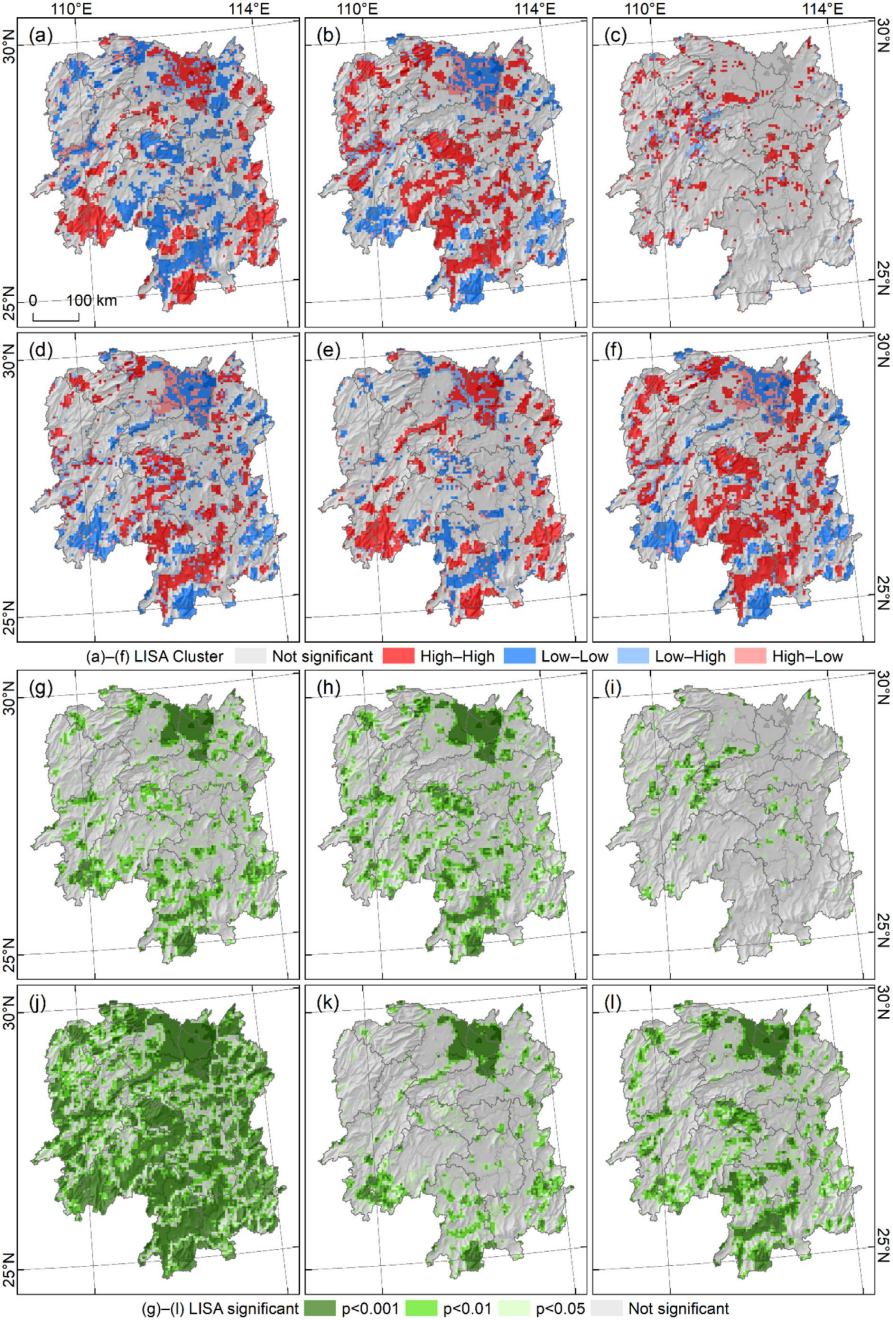
Bivariate local spatial autocorrelation of landscape index for LULC and habitat quality in Hunan Province in 2023. (a–f) Landscape index LISA distribution of LPI, ED, FRAC, PD, COHESION, and SHDI, respectively. (g–l) Landscape index LISA significant distribution of LPI, ED, FRAC, PD, COHESION, and SHDI, respectively.

The bivariate local spatial autocorrelation of the landscape indices of LULC and habitat quality in Hunan Province in 2023 demonstrated distinct clustering characteristics across different indices. All landscape indices exhibited four types of clustering patterns, including high–high, low–low, low–high, and high–low, and with high–high and low–low clustering showing the strongest statistical significance (Figure 12). The high–high clustering of LPI was primarily distributed in northern, southwestern, southern, and southeastern Hunan, while the low–low clustering was concentrated in central and southern Hunan (Figure 12a). The ED showed clustering patterns roughly opposite to those of LPI, with high–high clustering mainly located in northwestern and central southern Hunan, and low–low clustering mainly distributed in northern, southwestern, southern, and southeastern Hunan (Figure 12b). The FRAC exhibited relatively indistinct spatial clustering, with only sporadic high–high clustering in northwestern and central Hunan (Figure 12c). For PD, high–high clustering was concentrated in central and southern Hunan, while the low–low clustering was found in northern, southwestern, southern, and eastern regions (Figure 12d). The high–high clustering of COHESION was primarily distributed in northern, southwestern, southern, and southeastern Hunan, while the low–low clustering was in central and southern regions (Figure 12e). The SHDI's clustering patterns were highly similar to those of ED, with high–high clustering mainly located in northwest and central southern Hunan, and low–low clustering in northern, southwestern, southern, and southeast regions (Figure 12f). The significance distribution of Local Indicators of Spatial Association (LISA) was consistent with the clustering patterns, where stronger clustering corresponded to higher statistical significance, especially PD (Figure 12j).

## 4 Discussion

### 4.1 LULC Changes Characteristics Comparison and Driving Factors

The characteristics and spatial distribution of LULC in Hunan Province are closely associated with natural environmental factors, including elevation and NDVI distribution. The dominance of forestland in the province indicates a relatively favorable natural environment, particularly in the mountainous regions of western, southern, and eastern Hunan, where it is characterized by minimal human disturbance. Notably, high NDVI values are also concentrated in these mountainous regions, which aligns with the extensive distribution of forests observed here. The spatial distribution and temporal changes of LULC landscape indices further reveal increasing ecosystem fragmentation, reduced stability, and enhanced heterogeneity. These trends suggest that human interference has become increasingly prominent, especially in river valleys and plains regions conducive to socioeconomic activities. Consequently, LULC transfers are primarily characterized by the conversion of other land types to built‐up land, with rare reversals of this process. Additionally, the mutual conversion between cultivated land and forestland reflects both the development and restoration of land resources in the province.

Changsha, a typical region of rapid urbanization in Hunan Province, provides clear evidence of human‐driven LULC changes. From 2000 to 2020, socioeconomic factors (e.g., GDP, total agricultural output), topographic conditions (e.g., elevation, slope), and climate change (e.g., annual average temperature, annual precipitation) collectively drove a reduction in cultivated land, with conversion to forestland and built‐up land (Zhang, Lu, et al. 2024). Consistent with this, remote sensing monitoring has shown a significant increase in built‐up region density and urban expansion in Changsha from 1990 to 2001 (Huang et al. 2015). These findings confirm that urban land expansion is a key characteristic and trend of LULC change in the region, which also reflects the clustering of human activities. Dongting Lake, as a critical ecological hinterland of Hunan Province, experienced intense LULC conflicts from 2000 to 2020, with these conflicts concentrated in highly urbanized and densely populated regions (An et al. 2023). The Dongting Lake basin also exhibited the fastest transfer rate of built‐up land (Yang et al. 2021), highlighting the impact of human activities, especially urban expansion, on LULC structure.

The LULC changes characteristics observed in Hunan Province are more pronounced in typical urban agglomerations, and comparisons with China and other countries' cases further contextualize these trends. While the United States has a longer history of urbanization and its three major urban agglomerations cover vast areas with relatively mature development, the built‐up land area of China's three major urban agglomerations has exceeded that of their US counterparts in recent decades (He et al. 2019). Specifically, the Yangtze River Delta urban agglomeration in China exhibited concentrated patch expansion of built‐up land from 2000 to 2020, reflecting significant economies of scale; this expansion has increasingly eroded relatively intact farmland and forestland (Zhai et al. 2024). Similar prominent LULC change characteristics have been documented in both the Pearl River Delta urban agglomeration and the Wuhan Urban Agglomeration (Hu et al. 2021; Lu et al. 2014), raising critical considerations on sustainable development and the coordination of LULC conflicts (Bao et al. 2021).

### 4.2 Habitat Quality Changes Characteristics Comparison and Driving Factors

The spatial distribution of habitat quality in Hunan Province is closely linked to both natural environmental conditions and the intensity of human activities. The western, southern, and eastern parts of the province boast a favorable natural environment, characterized by good vegetation coverage and minimal human disturbance, which collectively support high habitat quality. In contrast, river valley plains and lake regions, being conducive to urban construction and agricultural development, experience concentrated human activities, resulting in relatively low habitat quality. The spatial distribution and temporal changes of temporal landscape indices further reveal increasing fragmentation, reduced stability, and enhanced heterogeneity of habitat quality across the province, all of which indicate an overall trend of habitat quality deterioration. This conclusion is further supported by the proportional changes and transfer dynamics of habitat quality, which consistently show a shift from relatively high‐quality to relatively low‐quality grades.

These findings align with results from related regional studies. For instance, the overall environmental quality of the Yangtze River Basin region (of which Hunan Province is a key component) decreased by 0.408% from 2000 to 2020, accompanied by a significant increase of 15.396% in human activities, and the flourishing human activities in the Yangtze River region pose a major threat to habitat restoration work, especially in the middle and lower reaches (Bian et al. 2024). Similarly, the habitat quality of the Xiangjiang River Basin (within Hunan Province) declined gradually during the 1990–2020 period (from 0.770 to 0.757), with high‐quality habitat primarily distributed in mountainous regions and habitat degradation concentrated in urban regions of the middle and lower reaches (Hong et al. 2023), which is similar to the results of the present study. Beyond the immediate study area, the Luoxiao Mountain region (spanning southeastern Hunan and southern Jiangxi Province) serves both as a critical ecological barrier and a concentrated poverty‐stricken zone. Guo, Niu, et al. (2023) found that the overall decline trend in habitat quality from 1995 to 2020 was driven by the combined effects of natural geographical factors (e.g., slope, precipitation) and socioeconomic factors (e.g., GDP). Even within national nature reserves, a decline trend in habitat quality has been found (Wang, Liu, Xie, et al. 2023), underscoring the urgency of strengthening ecological environment conservation efforts.

Notably, the decline in habitat quality in Hunan Province is not an isolated phenomenon. Similar habitat quality degradation has been documented in the Guangdong Hong Kong Macao Greater Bay Area, highly urbanized cities such as Shenzhen, the Yangtze River Delta urban agglomeration, and the Beijing Tianjin Hebei urban agglomeration. It has also been observed in mining areas, as well as in more ecologically sensitive regions such as natural wetlands and nature reserves. These widespread changes highlight the pressing need for enhanced habitat protection, as it is not only vital to maintaining human living environments but also critical for preserving biodiversity.

### 4.3 Comparison of the Response of Habitat Quality to LULC Changes

In Hunan Province, the response of total habitat quality to LULC changes is most pronounced in the mutual conversion between cultivated land and forestland, primarily because these two land types occupy the largest areas. From the perspective of average habitat quality, the mutual conversion between other land types and built‐up land contributes most significantly to variations in habitat quality. This is attributed to the substantial impact of built‐up land on habitat quality degradation, which in turn reflects the influence of human activities on ecosystems. Specifically, the conversion of cultivated land to forestland and built‐up land to other land types indicates a reduction in human interference with ecosystems, thereby making positive contributions to habitat quality. Contrary, the transfer of forestland to cultivated land and other land types to built‐up land reflect increased human efforts to transform the surface environment, resulting in negative contributions to habitat quality changes.

These findings are supported by, and contextualized within, related studies. For instance, habitat quality in the Dongting Lake Basin stayed relatively high from 1980 to 2018 but kept deteriorating; its response to different land types showed notable spatiotemporal heterogeneity, with forestland, grassland, and water bodies changes positively correlated with overall habitat quality, and farmland and built‐up land negatively correlated (Han et al. 2023). From 1985 to 2015, habitat quality around cities and roads in the Taihu Lake Basin declined severely, mainly due to urban expansion and farmland conversion to built‐up land (Xu et al. 2019). In Hebei's Taihang Mountains region (1990–2020), overall habitat quality was relatively high yet showed a downward trend, with changes significantly linked to land use, and areas dominated by human activity‐related built‐up land or cultivated land having lower habitat quality (Yang 2021). In Malaysia's estuarine delta (1989–2047), habitat quality also degraded, with main contributions from LULC changes like forest, agricultural land, residential land expansion, and land reclamation (Zhang, Yan, et al. 2024). Conversely, Zhangjiakou City in Hebei maintained high and improving habitat quality from 2000 to 2015; different land types had heterogeneous spatiotemporal impacts—forestland was generally positively correlated with habitat quality and had the greatest influence, grassland was roughly positively correlated with a north‐to‐south decreasing regression coefficient, and cultivated land and built‐up land were mainly negatively correlated with habitat quality spatially, with positive correlations in some areas (Wang et al. 2019). These studies indicate that there are differences in habitat quality changes in different regions. While existing studies have explored the impact of LULC changes on habitat quality changes (degradation), they remain insufficient in quantifying specific contributions. In contrast, the present study addresses this gap by conducting a quantitative analysis of the contributions of different LULC conversions to changes in habitat quality in Hunan Province, providing more precise insights into the mechanisms driving regional habitat quality dynamics.

### 4.4 Spatial Correlation Between Landscape Index of LULC and Habitat Quality

In Hunan Province, strong correlation, bivariate global spatial positive correlation, and significant spatial clustering characteristics were observed between the landscape indices of LULC and habitat quality. Specifically, the LPI, ED, PD, COHESION, and SHDI collectively indicate that increased fragmentation, reduced stability, and enhanced heterogeneity of LULC can trigger significant and analogous changes in habitat quality. However, the FRAC exhibited insignificant effectiveness, highlighting the need to select appropriate landscape indices based on specific research objectives.

The analysis of LULC landscape indices characteristics and the exploration of their relationships with habitat quality have garnered widespread attention in existing studies. For instance, the InVEST model combined with landscape pattern indices has been applied to evaluate mountain plant biodiversity changes (Gong et al. 2019). Studies across different regions have revealed distinct links between landscape indices and habitat quality. In the Yangtze River Economic Belt (2000–2030), landscape indices show improved land use level (via Shannon's diversity index), higher fragmentation, and lower connectivity, with strong local spatial autocorrelation between these indices and habitat quality, alongside significant spatiotemporal heterogeneity (Yang, Yang, et al. 2024). In the Yellow River Basin, the PD and SHDI increased notably, while the ED, LS, average patch area (AREA_MN), and CONTAG decreased, with habitat quality showing negative spatial relationships with PD and COHESION and positive impacts from ED and LSI (Zhang et al. 2022). In the Guanzhong Plain urban agglomeration (2000–2015), landscape indices changed significantly (trending toward fragmentation, with more diverse and uniform types), and habitat quality had clear spatial correlation with landscape pattern, with obvious clustered spatial differentiation (Gu et al. 2023). Additionally, rising urbanization may weaken the clustering effect of habitat quality and landscape pattern characteristics. In Northeast China (1990–2010), LULC changes reduced landscape connectivity and increased fragmentation, agricultural land contributed significantly to habitat degradation, and patch density and fragmentation index had a clear negative correlation with habitat quality index (Dai et al. 2019). While these studies also focus on the impact of landscape patterns on habitat quality, the application of landscape indices remains insufficient. Notably, habitat quality itself constitutes a geographical element, and landscape indices can also be utilized to analyze the spatiotemporal variation characteristics of habitat quality. Therefore, the present study employed landscape indices to analyze the characteristics of LULC and habitat quality, while further exploring their spatial correlations.

### 4.5 Limitations and Prospects

This study analyzed the habitat quality of Hunan Province in South China and its response to LULC changes. However, there are some shortcomings in this study: (1) We adopted the widely used InVEST model, which facilitates comparative analysis between different studies. However, the applicability of other methods and the comparison of results with other methods in this study region were not considered. Therefore, comparisons between different methods on the same topic should be considered. (2) We have explored the response of habitat quality to land use change based on clustering characteristics and spatial autocorrelation, which is only preliminary. Therefore, more methods need to be incorporated, such as using deep learning methods to quantify the contributions of different LULCs, using geographically weighted regression to quantify the spatial distribution of contributions, and using geographic detectors to quantify the contribution of driving factor interactions to habitat quality changes. (3) This study mainly analyzed the response of habitat quality changes to LULC changes, but the reasons for habitat quality changes are complex. Therefore, multiple driving factors need to be considered, including the combined impact of natural environment and socioeconomic factors.

## 5 Conclusion

This study evaluated changes in habitat quality in Hunan Province using the InVEST model, with the main conclusions drawn as follows: The LULC in Hunan Province is dominated by forestland (62.16%–62.6%) and cultivated land (27.82%–28.83%). Forestland is primarily distributed in the mountainous regions of western, southern, and eastern Hunan, while cultivated land is mainly concentrated in the middle and lower reaches of rivers, plains, and around lake regions in central and northern Hunan. Other LULC types are mostly scattered in a dotted pattern. The spatial distribution and temporal changes of LULC landscape indices indicate increased ecosystem fragmentation, reduced stability, and enhanced heterogeneity. LULC transfers are dominated by the mutual conversion between cultivated land and forestland, with other land types mainly converted to built‐up land, particularly prominent during the 2015–2020 period. The habitat quality in Hunan Province is mainly categorized as high‐quality (40.73%–42.23%), higher‐quality (22.58%–23.56%), and medium‐quality (24.14%–25.1%), which are primarily distributed in the mountainous regions of southwestern, southern, and eastern Hunan, as well as around lakes in northern Hunan. The spatial distribution and temporal changes of habitat quality landscape indices reflect increased ecosystem fragmentation, reduced stability, and enhanced heterogeneity, indicating an overall trend of habitat quality deterioration. Habitat quality transfers are mainly characterized by shifts from relatively higher habitat quality to relatively lower habitat quality grades, with the most frequent transfers occurring between 2015 and 2020. Habitat quality changes in Hunan Province from 2000 to 2023 were primarily driven by the mutual transfer of cultivated land and forestland, as well as the mutual transfer of built‐up land and other land. For total habitat quality changes, the most significant contributions came from the conversion of cultivated land to forestland (29.86%) and forestland to cultivated land (30.26%), which exerted positive and negative effects, respectively. For average habitat quality changes, the most prominent driver was the mutual transfer between built‐up land and other land types. The transfer of other land types to built‐up land mainly made negative contributions (exceeding 4.93%), while the conversions from built‐up land to other land types primarily made positive contributions (exceeding 4.93%). Strong correlations, bivariate global spatial positive correlations, and significant spatial clustering characteristics were observed between LULC landscape indices and habitat quality landscape indices. Specifically, the LPI, ED, PD, COHESION, and SHDI collectively indicate that increased fragmentation, reduced stability, and enhanced heterogeneity of LULC can trigger significant and analogous changes in habitat quality.

The findings of this study highlight that land use planning should prioritize the conservation of high‐quality habitats, particularly in mountainous regions (e.g., western Hunan and southern China) as well as riparian and lacustrine areas. Stringent measures are required to regulate the unordered conversion of cropland and forestland, thereby preventing the encroachment of construction land on high‐quality habitat patches. Furthermore, a dynamic monitoring mechanism should be established to quantitatively constrain the intensity of LULC transitions. Multimethod integrated assessments of habitat quality and its driving factors are also recommended, which can provide robust scientific support for ecological civilization construction and regional sustainable development.

## Author Contributions

**Xiaojun Wang:** conceptualization (equal), data curation (equal), methodology (equal), resources (equal), software (equal), validation (equal), visualization (equal), writing – original draft (equal), writing – review and editing (equal). **Guangxu Liu:** conceptualization (equal), data curation (equal), project administration (equal), resources (equal), writing – original draft (equal), writing – review and editing (equal). **Hong Jia:** conceptualization (equal), data curation (equal), methodology (equal), resources (equal), software (equal), writing – original draft (equal).

## Ethics Statement

The authors have nothing to report.

## Consent

The authors have nothing to report.

## Conflicts of Interest

The authors declare no conflicts of interest.

## Data Availability

The land use/cover data derived from the Resources and Environment Science Data Center of the Chinese Academy of Sciences (https://www.resdc.cn/), and the DEM data derived from the SRTM (https://www.earthdata.nasa.gov/data/instruments/srtm).
